# Unveiling Secondary Mutations in Blended Phenotypes: Dual ERCC4 and OTOA Pathogenic Variants Through WES Analysis

**DOI:** 10.3390/ijms252413471

**Published:** 2024-12-16

**Authors:** Pinella Failla, Lucia Saccuzzo, Ornella Galesi, Donatella Greco, Vincenza Barresi, Silvestra Amata, Corrado Romano, Marco Fichera

**Affiliations:** 1 Oasi Research Institute-IRCCS, via Conte Ruggero 73, 94018 Troina, Italy; pfailla@oasi.en.it (P.F.); ogalesi@oasi.en.it (O.G.); dgreco@oasi.en.it (D.G.); samata@oasi.en.it (S.A.); 2 Department of Biomedical and Biotechnological Sciences, Section of Clinical Biochemistry and Medical Genetics, University of Catania, via Santa Sofia, 95123 Catania, Italy; lrsa@live.it (L.S.); corrado.romano@unict.it (C.R.); marco.fichera@unict.it (M.F.); 3 Research Unit of Rare Diseases and Neurodevelopmental Disorders, Oasi Research Institute-IRCCS, via Conte Ruggero 73, 94018 Troina, Italy

**Keywords:** *ERCC4*, *OTOA*, run of homozygosity, whole exome sequencing, blended phenotypes, dual diagnoses

## Abstract

This study describes two siblings from consanguineous parents who exhibit intellectual disability, microcephaly, photosensitivity, bilateral sensorineural hearing loss, numerous freckles, and other clinical features that suggest a potential disruption of the nucleotide excision repair (NER) pathway. Whole exome sequencing (WES) identified a novel homozygous missense variant in the *ERCC4* gene, which was predicted to be pathogenic. However, a subsequent peculiar audiometric finding prompted further investigation, revealing a homozygous deletion in the *OTOA* gene linked to neurosensorial hearing loss. Both variants were located within a run of homozygosity (ROH) on chromosome 16p13.12-p12.2, implicating a complex genetic basis for the observed phenotype. While this study reports a potentially novel *ERCC4* variant, it underscores the importance of comprehensive analysis and deep phenotyping in WES data to improve diagnostic accuracy. Our findings advocate for an expanded approach in WES analysis, ensuring more precise diagnoses and improved genetic counseling, particularly when specialized tests for structural variant analysis are unavailable.

## 1. Introduction

Emerging evidence clearly indicates that a significant portion of genetic diseases is driven by deleterious variants occurring at multiple loci [1]. The resulting blended phenotypes depend on the specific genes affected and can be broadly classified into two categories: distinct—when the genes are associated with different clinical features, or overlapping—when the locus-specific phenotypes share some or most clinical traits [2]. Intuitively, in cases with overlapping phenotypes, the identification of all causative variants may be elusive. This is particularly true for genetic tests that target a limited number of genes or specific classes of variants. Consequently, a positive result from such tests may lead to an incomplete initial genetic diagnosis, potentially hindering further investigations.

For example, a physician might choose to conclude genetic testing after obtaining a positive, yet not entirely conclusive, result from a DNA microarray or WES test. This decision could potentially lead to overlooking an additional causative single nucleotide variant or a copy number variant that might fully explain the patient’s phenotype. Clearly, an incomplete genetic diagnosis can significantly impact genetic counseling, recurrence risk assessment, and, potentially, the appropriate therapies.

Dual locus pathogenic variants in patients with healthy parents can arise from various segregation combinations, including two de novo dominant pathogenic variants, a de novo variant plus two inherited recessive variants, or biallelic recessive variants at two loci. The latter combination is more common in consanguineous families than in outbred families due to the inheritance of identical segments from a common ancestor [3,4]. Consequently, the rarer co-occurrence of two recessive diseases in the same patient is expected to be more prevalent among consanguineous.

Here, we report a complex phenotype in two affected siblings from consanguineous parents, characterized by intellectual disability, microcephaly, photosensitivity, widespread freckles, bilateral sensorineural hearing loss, flexion contractures, and short stature. These features suggest a disease within the spectrum of disorders associated with defects in the nucleotide excision repair (NER) pathway. The NER pathway is essential for maintaining genomic integrity, particularly in repairing DNA damage caused by UV radiation and other environmental factors. Defects in components of the NER pathway predispose individuals to a range of diseases, which are characterized by a complex combination of clinical features, including photosensitivity, neurological degeneration, neurosensorial hearing loss, and an elevated risk of cancer [5].

Further clinical evaluation two years later, while confirming the initial clinical suspicion, also evidenced in both brothers a peculiar audiometric test showing mid-frequency hearing loss, not characteristic of NER-defective patients. This raised the possibility of dual-locus involvement and prompted us to conduct an in-depth WES analysis, searching for causative single nucleotide variants (SNVs), small insertions/deletions, and employing additional bioinformatic tools to identify copy number variants (CNVs) and runs of homozygosity (ROHs). This approach enabled us to identify two distinct loci with different molecular alterations, potentially contributing to the phenotype of our patients. Beyond this specific case, we discuss the importance of deep phenotyping and gathering as much information as possible from WES data to enhance the clinical sensitivity of genetic testing.

## 2. Detailed Case Description

### 2.1. Clinical Data

**Figure 1:**
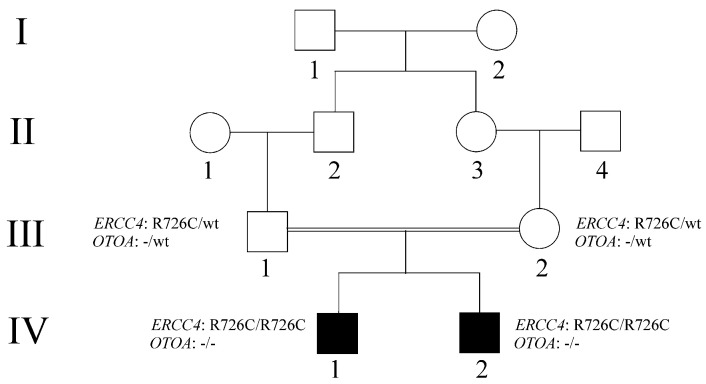
Pedigree of the extended family. Black-filled boxes represent affected individuals with a clinical presentation suggestive of a defect in the NER pathway and carry homozygous variants in both ERCC4 and OTOA. The parents (III−1 and III−2) are healthy individuals heterozygous for both the ERCC4 and OTOA variants.

**Figure 2:**
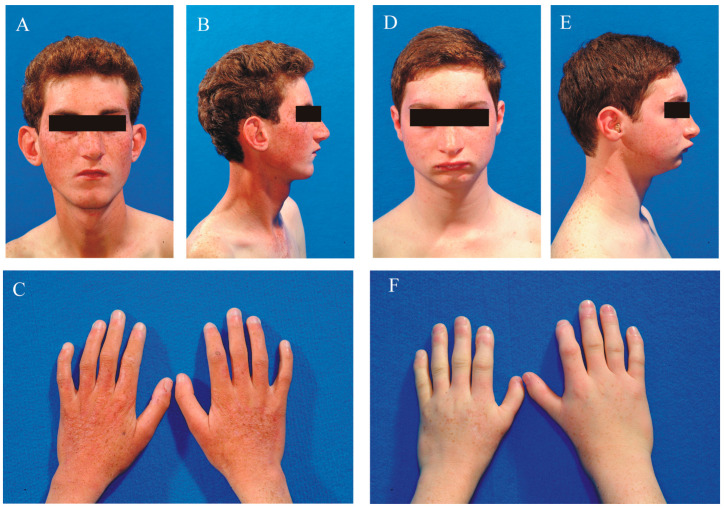
Clinical appearance of the affected brothers. Panels (**A**–**C**) depict patient IV−1, while panels (**D**–**F**) show patient IV−2. Numerous freckles are visible on the face, shoulders, and hands of both patients.

Parental informed consent was obtained for this study. The family pedigree is shown in Figure 1. The parents (III-1 and III-2), who are first cousins, are both healthy. Patient iv-1 (Figure 2A–C) is a male born at term via eutocic delivery following a pregnancy complicated by first-trimester abortion threats. At birth, he was small for gestational age, weighing 2020 grams, with a length of 43 cm and an occipital-frontal circumference (OFC) of 28.2 cm. He presented with generalized hypotonia, feeding difficulties, and delays in motor and language development, walking at 18 months and speaking his first word at 30 months.

His gait has always been wide-based with flexed knees and trunk, and he exhibited limitations in elbow joint movements. Until the age of 4, he experienced seizures without fever. A brain CT scan performed at age 8 revealed agenesis of the corpus callosum and dilation of the lateral ventricles, while the EEG was normal. Throughout childhood, he experienced severe photosensitivity and developed widespread freckles. During school age, he was diagnosed with learning difficulties and moderate-to-severe intellectual disability. He also presented growth delays and weight deficits.

At 23 years of age, he exhibited an uncertain, wide-based gait, limited language abilities restricted to simple sentences, and moderate intellectual disability. Photosensitivity and freckles have persisted since childhood. He was diagnosed with sensorineural hearing loss at 24 years, primarily affecting the mid frequencies, and suffers from allergic asthma with an obstructive component.

His clinical phenotype includes microcephaly, short stature, weight deficit, hypotonia with limb flexion, scoliosis, widespread freckles on the face, trunk, hands, and genitalia, scleral telangiectasias, and hearing loss. His facial appearance is characterized by a wizened expression, sunken eyes, photosensitivity, and skin that appears wrinkled and aged. He also demonstrates a progressive loss of facial subcutaneous fat. Brain MRI confirmed agenesis of the corpus callosum and dilation of the lateral ventricles, while the EEG was abnormal.

Patient IV-2 (Figure 2D–F) is a male born at term via eutocic delivery following an uneventful pregnancy. At birth, he was small for gestational age, weighing 2000 g, with a length of 47 cm and an occipital-frontal circumference (OFC) of 31 cm. At age 4, he exhibited normal motor development but showed language delay and failure to thrive. Behavioral assessments were unremarkable, and his mood was euthymic. A brain MRI at that time was normal, and the EEG was within normal limits. Learning difficulties became evident during his school years.

At 15 years of age, he presented with mild intellectual disability and expressive language delay. Since childhood, he has experienced moderate photosensitivity and developed widespread freckles. He did not experience epileptic seizures. At age 16, similar to his brother, he was diagnosed with sensorineural hearing loss, predominantly affecting the mid frequencies. Additionally, he suffers from allergic asthma with an obstructive component.

His clinical phenotype is characterized by microcephaly, short stature, mild hypotonia, photosensitivity, widespread freckles on the face, trunk, hands, and genitals, scleral telangiectasias, hearing loss, a wizened appearance, and skin that appears aged. Both brain MRI and EEG findings were normal.

### 2.2. WES Analysis

Whole-exome sequencing (WES) was performed by an external service provider (Biodiversa, Rovereto (TN), Italy). According to the provider’s description, whole-exome enrichment was carried out using the Agilent Sure Select Human All Exon V6 + UTRs kit (Agilent Technologies, Santa Clara, CA, USA) following the kit’s recommendations and sequenced with the Illumina Novaseq sequencer (Illumina, San Diego, CA, USA) to generate 150 bp paired-end reads.

Data from FASTQ files were mapped to the human reference genome (UCSC GRCh37/hg19) using BWA-MEM software version 0.7.17, and duplicate reads were removed using Picard.

Base quality score recalibration, indel realignment, and variant calling were performed using GATK tools version 4.6.0.0 (https://gatk.broadinstitute.org/hc/en-us).

Variants were annotated using the VEP version 111 and Gemini version 0.20.1 software tools and then filtered according to their predicted effects and allele frequencies in the available public databases gnomAD (https://gnomad.broadinstitute.org/), 1000 Genomes (https://www.internationalgenome.org/), ESP6500 (https://krishna.gs.washington.edu/download/CADD/v1.3/ESP6500SI_inclAnno.tsv.gz), dbSNP (https://www.ncbi.nlm.nih.gov/snp/), and ClinVar (https://www.ncbi.nlm.nih.gov/clinvar/). Variants were prioritized either assuming an autosomal recessive or X-linked pattern of inheritance and classified according to the ACMG criteria [6] using VarSome version 12.7.0 (https://varsome.com/). Candidate variants were validated by the Integrative Genomics Viewer (IGV) [7] and technically verified by Sanger sequencing.

WES analysis was performed in both siblings (IV-1 and IV-2). The average coverage of the Refseq coding regions was 115× , with 85% of the bases covered at least 30× and 97% of the bases covered at least 10×. Given the overlapping phenotypes of the two brothers, the analysis was specifically focused on autosomal recessive and X-linked segregation patterns.

The analysis identified in the 16p13.12 region, a homozygous missense variant (NM_005236.3:c.2176C>T;p.Arg726Cys) involving ERCC4, coding for the XPF endonuclease—a key protein in the nucleotide excision repair (NER) pathway which is classified as a disease gene in the Online Mendelian Inheritance in Man (OMIM) catalog (MIM: 133520).

The variant was found in the genome Aggregation Database (gnomAD) with an extremely low frequency (gnomAD max: 0.000054) and was absent in the homozygous state. This SNV is also documented in the ClinVar database (VCV000843239.7) and is classified as a variant of uncertain clinical significance (VUS).

**Figure 3:**
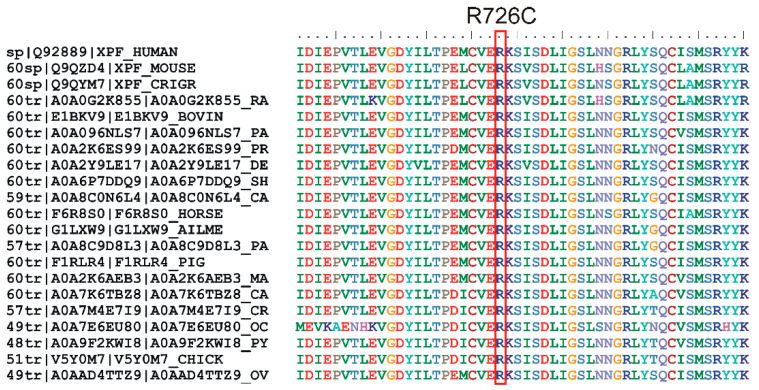
Multiple sequence alignment of homologous *ERCC4* proteins from various species (generated via UniProt), demonstrating the evolutionary conservation of the arginine residue at position 726 in human *ERCC4* (highlighted in a red box). The high conservation of this amino acid suggests its functional or structural importance, indicating selective pressure to preserve this residue across diverse species.

The missense change was predicted to be deleterious and considered destabilizing at the protein level by several prediction software tools (Table 1). The variant was classified by VarSome as VUS. Phylogenetic analysis demonstrated that the arginine at position 726 was highly conserved, even in the evolutionarily distant species (Figure 3).

Sanger sequencing confirmed the presence of the variant in both homozygous siblings and in their heterozygous parents.

No additional candidate variants were identified through routine WES analysis. Most of the clinical features observed in our patients, along with the homozygous *ERCC4* gene variant, are consistent with Xeroderma Pigmentosum, Type F/Cockayne Syndrome, one of the disorders associated with *ERCC4* alterations [8], although without the typical development of skin cancer (as shown in Table 2). However, the hearing loss usually associated with *ERCC4* variants typically affects high frequencies, while our patients experienced mid-frequency hearing deficits. This discrepancy prompted us to investigate further for other potentially pathogenic variants that might have been missed in the routine WES analysis.

### 2.3. Excavator2 Analysis

The core algorithm behind Excavator2 [9], a tool designed for detecting copy number variants (CNVs) from WES data, is a Heterogeneous Hidden Markov Model (HMM), which is used for segmenting and identifying CNVs. Additionally, the software employs the Shifting Level Model (SLM) for segmenting normalized read counts (RC), incorporating the distance between consecutive exons to enhance detection accuracy across varying genomic regions.

**Figure 4:**
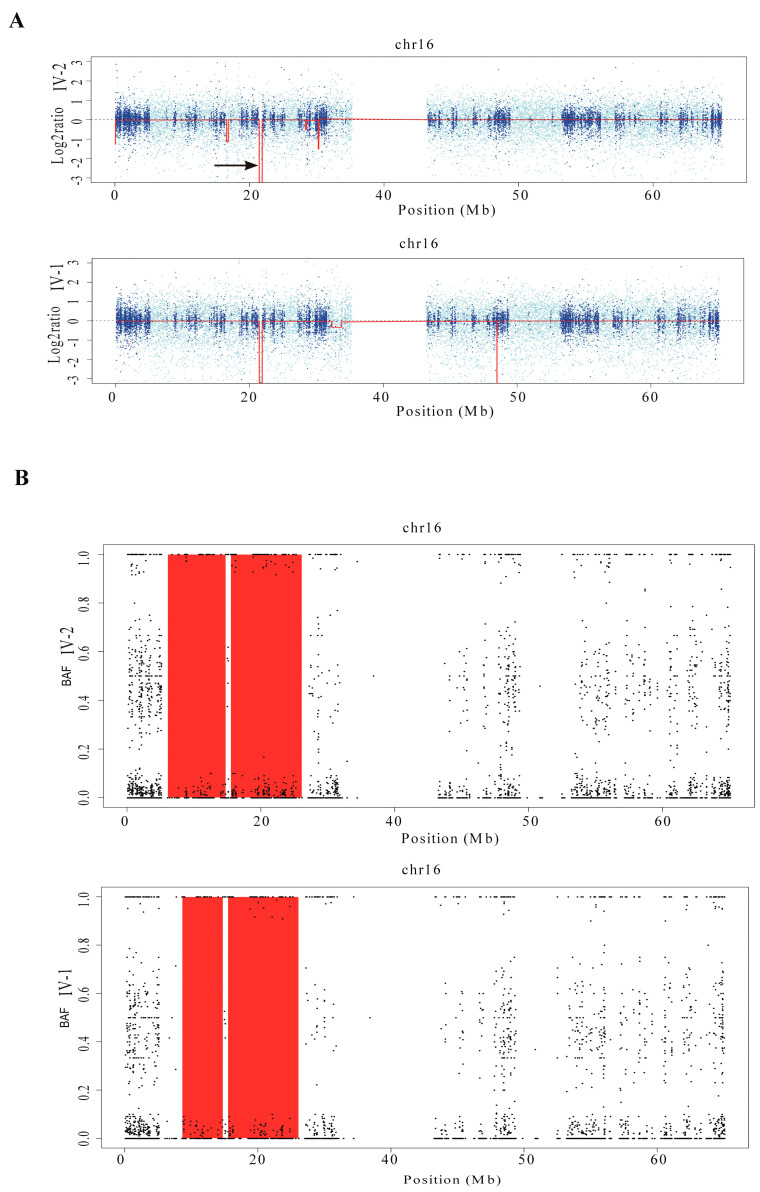
Chromosome 16 analysis of CNVs and ROHs using WES data. (**A**) Arrows indicate a homozygous deletion of approximately 317 kb (chr16: 21,561,888−21,878,568), detected in both siblings using Excavator2 software, which includes the *OTOA* gene. (**B**) B-allele frequency (BAF) analysis using H3M2 revealed that both siblings share an approximately 17 Mb ROH on chromosome 16p13.3-p12.1, encompassing both the deletion involving *OTOA* and the missense variant in *ERCC4*.

Copy number variants (CNVs) were identified from WES data using the Excavator2 software. The analysis detected a homozygous deletion estimated at approximately 317 kb (chr16: 21,561,888–21,878,568) in both siblings (Figure 4A). This deletion overlaps with a recurrent CNV linked to non-allelic homologous recombination between segmental duplications BP1 and BP2 on chromosome 16p12.2. The affected region includes the genes *METTL9* (MIM:609388), *IGSF6* (MIM:606222), and *OTOA* (MIM:607038), the latter of which is associated with autosomal recessive deafness syndrome DFNB22 (MIM:607039).

### 2.4. H3M2 Analysis

The H3M2 software (https://sourceforge.net/projects/h3m2/, accessed on 10 December 2024), leveraging the Heterogeneous Hidden Markov Model, provides a tool for detecting LOH from WES [10]. The tool comprises two main modules: the Bam Parsing Module, which is responsible for generating the B-allele frequency (BAF) values from the input BAM files, and the Analysis Module, which identifies LOHs from BAF values. As suggested by the authors, the running parameters Dnorm, P1, P2, and factor were set to 100,000, 0.1, 0.1, and 5, respectively.

Loss-of-heterozygosity (LOH) analysis using the H3M2 tool was conducted on BAM files from both siblings. The BAF analysis in the probands identified extensive LOH regions totaling 197 Mb. According to the Excavator2 results, all identified regions were classified as ROHs, resulting in a calculated inbreeding coefficient (F) of 0.06. This value is consistent with the inbreeding levels commonly seen in the offspring of first-cousin parents.

Notably, both siblings shared a 17 Mb ROH on chromosome 16p13.3-p12.1 (Figure 4B), encompassing both the homozygous deletion in *OTOA* and the missense variant in *ERCC4.*

### 2.5. CGH/SNP ARRAY Analysis

Genomic DNA was extracted by standard procedures. CGH/SNP array assays were carried out using the SurePrint G3 Custom CGH Microarray, 2 × 400 K (Agilent Technologies, Santa Clara, CA, USA) according to the manufacturer’s protocol version 7.1, using appropriate Agilent Reference DNAs (Euro male and Euro female). The arrays were analyzed with the Agilent Microarray Scanner, Feature Extraction Software version 11.5, and Agilent Genomic Workbench 7.0.

**Figure 5:**
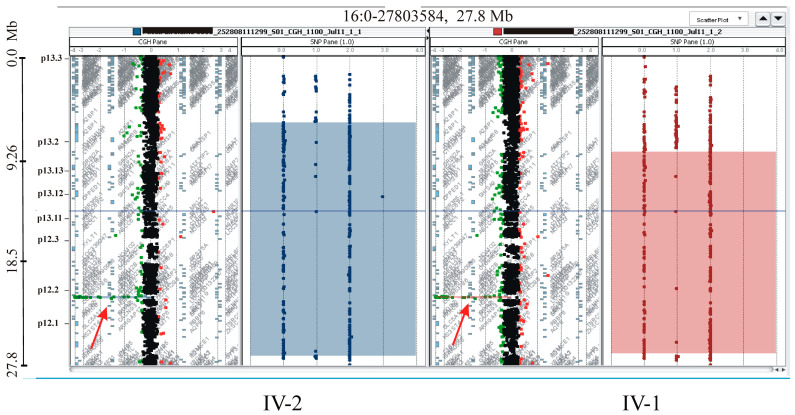
Array-CGH/SNP analysis of patients. The **left panel** for each patient displays the log2 ratios along chromosome 16, with red arrows indicating the 180 kb homozygous deletion involving *OTOA*. The shadow boxes on the **right panels** highlight the homozygous regions on chromosome 16.

The CGH/SNP array analysis in both siblings and their parents confirmed and refined the deletion and ROH regions previously identified by Excavator2 and H3M2 (Figure 5). Specifically, the deletion size was estimated to be approximately 180 kb in both patients (arr[GRCh37] 16p12.2 (21559687_21739911)x0 mat pat). The ROH on chromosome 16 was redefined to approximately 18 Mb in patient IV-I (ISCN 2020: arr[GRCh37] 16p13.2p12.1(8325041_26952730)x2 hmz) and approximately 21 Mb in patient IV-2 (ISCN 2020: arr[GRCh37] 16p13.3p12.1(6000729_26952730)x2 hmz), respectively.

## 3. Discussion

We describe two brothers born to consanguineous parents, presenting with a clinical phenotype suggestive of a defect in the NER pathway. WES analysis identified a novel homozygous missense variant in the *ERCC4* gene (MIM:133520), located on chromosome 16p13.12, supporting the diagnosis. *ERCC4* encodes the endonuclease XPF, which forms a complex with ERCC1 to participate in nucleotide excision repair (NER), specifically catalyzing the 5′ incision during DNA repair [11].

Pathogenic variants in *ERCC4* are associated with disorders such as Xeroderma pigmentosum group F (MIM:278760), Cockayne Syndrome (MIM:216400), a combined form known as XPF/CS (MIM:278760), Fanconi anemia (FANQ) (MIM:615272), and XFE progeroid syndrome (MIM:610965) [11,12,13,14,15]. Rare cases exhibit combined features of these syndromes [14]. An up-to-date summary of reported ERCC4 patients, along with their main clinical signs, is available in Supplementary Table S1.

While high-frequency sensorineural hearing loss is common in ERCC4-associated diseases [16], our patients showed significant mid-frequency hearing loss (750 to 2000 Hz). This anomaly suggested an additional locus might be involved, prompting a reanalysis of WES data for LOHs and CNVs. Using Excavator2 software, we identified a homozygous deletion in the *OTOA* gene. Concurrently, H3M2 software revealed a shared 17 Mb region of homozygosity on chromosome 16p13.3-p12.1, encompassing both *OTOA* and *ERCC4*, which was confirmed by array-CGH/SNP analysis.

The *OTOA* gene (MIM:607038) on chromosome 16p12.2 encodes otoancorin, which is crucial for inner ear function [17]. Mutations in *OTOA* cause autosomal recessive deafness DFNB22 (MIM:607039), which typically presents with mid-frequency hearing loss [18].

Our findings indicate that the complex phenotype in these patients may result from co-segregation of pathogenic variants in two distinct genes on chromosome 16, indicating a potential dual diagnosis having significant implications for genetic counseling and recurrence risk assessment. It is noteworthy that while the primary clinical features in these brothers are attributable to ERCC4 variants, the observed clinical variability, such as the agenesis of the corpus callosum (Table 2), suggests a more intricate interplay between genetic and non-genetic factors. Recent studies have increasingly highlighted that many diseases previously considered to follow simple Mendelian inheritance are, in fact, shaped by a combination of modifier genes, epigenetic influences, and environmental factors [19,20]. In this case, despite both brothers harboring the same primary mutations, differences in modifier genes, epigenetic regulation, or prenatal and postnatal environmental exposures may contribute to the phenotypic divergence, thereby adding further complexity to our understanding of this disorder.

Despite our findings, this study has several limitations. We acknowledge that interpreting the functional roles of novel candidate variants—particularly missense or non-coding regulatory variants—requires caution. In the context of consanguinity, such variants may frequently appear in a homozygous state solely due to shared ancestry rather than because they effectively contribute to the observed phenotype. This raises the possibility that they may represent ultra-rare or family-specific benign variants, possibly leading to misdiagnosis. While the homozygous deletion in OTOA is a well-established cause of deafness, the pathogenicity of the *ERCC4* variant identified in our patients is currently supported only by allele frequency data, in silico predictions, and clinical concordance. As this variant lacks functional validation and has not been reported in other patients, further studies are necessary to fully elucidate its potential pathogenic role.

These cases may add to reports of patients whose complex phenotypes result from the simultaneous involvement of multiple genes [2,21,22,23]. Such occurrences are estimated in about 5% of patients undergoing WES [1], likely an underestimate due to undetected or hard-to-interpret variants.

Genetic diagnosis is complex due to factors like genetic heterogeneity, overlapping symptoms, and various genetic alterations requiring multiple tests. Thorough clinical assessment is crucial to guide appropriate diagnostic testing and ensure that positive results fully explain all clinical features. However, this task can be particularly challenging for rare diseases, many of which may present as spectrum disorders.

In all cases, a deep characterization of the phenotype is a crucial step toward a comprehensive diagnostic workflow. Indeed, in our patient, the detailed clinical description enabled a dual diagnosis by distinguishing between mid-frequency and high-frequency hearing loss. While WES and targeted gene panels are often first-tier tests for suspected Mendelian diseases, they may miss structural variants. We recommend using bioinformatic tools to analyze WES data for CNVs and uniparental disomy to identify potential dual diagnoses without additional laboratory work.

## 4. Conclusions

Today, we are on the threshold of incorporating new analysis techniques based on long-read sequencing into clinical diagnostics. Alongside the new, gapless, T2T human genome reference, this will allow for the characterization of the entire spectrum of genomic variants in a single test. While facilitating the identification of individuals with multiple diagnoses, this will significantly shift the focus from the identification of variants to understanding their role in clinical presentation [24]. In this context, multiple diagnoses represent just the tip of the iceberg in a complex interplay of high-risk factors, modifier genes, hypomorphic alleles, and environmental influences that shape the overall clinical picture of our patients.

## Figures and Tables

**Table 1:** In silico pathogenicity prediction of the *ERCC4*;p.Arg726Cys variant.

**Software**	**Score**	**Prediction**
SIFT v6.2.1	0	deleterious
Polyphen-2	1	probably damaging
CADD 1.7	28	moderate pathogenic
REVEL v1.3	0.774	likely disease-causing
MetaLR v0.14.7	0.584	damaging
Mutation Assessor v4	0.965	high pathogenic
AlphaMissense v2.0	0.968	deleterious
**Protein stability**	**ΔΔG (kcal/mol)**	
MUpro v1.0	−0.883	destabilizing
MAESTROweb v1.2.35	0.184	neutral
DynaMut v2	−0.97	destabilizing
DDGun v2	−1.3	destabilizing
mCSM	−1.907	destabilizing
CUPSAT	−1.68	destabilizing

The pathogenicity prediction follows the guidelines established by the authors. In the protein stability analysis, a negative ΔΔG indicates a decrease in protein stability, with values below −0.5 kcal/mol considered to be significantly destabilizing. Software links: SIFT (https://sift.bii.a-star.edu.sg/), Polyphen-2 (http://genetics.bwh.harvard.edu/pph2/), CADD (https://cadd.gs.washington.edu/), REVEL (https://sites.google.com/site/revelgenomics/), MetaLR (https://github.com/cnodes/metalr/), Mutation Assessor (http://database.liulab.science/dbNSFP), AlphaMissense (https://alphamissense.hegelab.org/search), MUpro (https://mupro.proteomics.ics.uci.edu), MAESTROweb (https://pbwww.services.came.sbg.ac.at/maestro/web/maestro), DynaMut2 (https://biosig.lab.uq.edu.au/dynamut2/), DDGun (https://folding.biofold.org/ddgun/), mCSM (https://biosig.lab.uq.edu.au/mcsm/), CUPSAT(https://cupsat.brenda-enzymes.org/).

**Table 2:** Correlation of the main clinical features of Xeroderma Pigmentosum, Type F/Cockayne Syndrome with the clinical presentation of the patients.

Clinical Features	Patient IV-1	Patient IV-2
Abnormality of the eye	no	no
Abnormality of the integument		
Cutaneous photosensitivity	yes	yes
Freckling	yes	yes
Abnormality of the musculoskeletal system		
Flexion contracture	yes	yes
Microcephaly	yes	yes
Scoliosis	yes	no
Abnormality of the nervous system		
Cerebellar ataxia	no	no
Dementia	no	no
Intellectual disability	yes	yes
Morphological central nervous system abnormality	Agenesis of the corpus callosum, dilation of the lateral ventricles	no
Hyperreflexia	yes	yes
Hearing impairment	yes	yes
Growth abnormality		
Decreased body weight	yes	yes
Short stature	yes	yes
Neoplasm	no	no

## Data Availability

The raw data supporting the conclusions of this article will be made available by the authors on request.

## Supplementary Materials

The following supporting information can be downloaded at: https://www.mdpi.com/article/10.3390/ijms252413471/s1. References [11,14,15,25,26,27,28,29,30,31,32,33,34] are cited in the supplementary materials.
